# Application of Tissue Microarray Technology to Stem Cell Research

**DOI:** 10.3390/microarrays3030159

**Published:** 2014-06-26

**Authors:** Alberto La Spada, Barnaba Rainoldi, Andrea De Blasio, Ida Biunno

**Affiliations:** 1 UOS-IRGB-CNR, Via Fantoli 16/15, 20138, Milano, Italy; E-Mail: laspada.alberto@gmail.com; 2 IRCCS-Multimedica, Via Fantoli 16/15, 20138, Milano, Italy; E-Mail: barnaba.rainoldi@multimedica.it; 3 Integrated Systems Engineering, Via Fantoli 16/15, 20138, Milano, Italy; E-Mail: andrea.deblasio@isenet.it

**Keywords:** tissue microArray (TMA), cell microArray (CMA), stem cells

## Abstract

There is virtually an unlimited number of possible Tissue Microarray (TMA) applications in basic and clinical research and ultimately in diagnostics. However, to assess the functional importance of novel markers, researchers very often turn to cell line model systems. The appropriate choice of a cell line is often a difficult task, but the use of cell microarray (CMA) technology enables a quick screening of several markers in cells of different origins, mimicking a genomic-scale analysis. In order to improve the morphological evaluations of the CMA slides we harvested the cells by conventional trypsinization, mechanical scraping and cells grown on coverslips. We show that mechanical scraping is a good evaluation method since keeps the real morphology very similar to those grown on coverslips. Immunofluorescence images are of higher quality, facilitating the reading of the biomarker cellular and subcellular localization. Here, we describe CMA technology in stem cell research.

## 1. Introduction

Immunohistochemistry and fluorescence *in situ* hybridization (FISH) are commonly applied to formalin-fixed, paraffin-embedded tumor tissues in diagnostic and in basic research laboratories [1]. However, the recent use of high throughput technical approaches to analyse molecular alterations occurring during cancer development (*i.e.*, expression and CGH arrays, protein analyses, *etc*.) have produced enormous pools of preliminary data that needs, eventually, to be validated. In order to define their clinical significance, large scale and well characterized clinical specimens need to be processed and screened for multiple markers, but the use of conventional approaches is tedious and time/costs-consuming. The application of tissue microarrays (TMA) platforms facilitates such analysis, indeed it is a very useful tool to bridge the gap between candidate discovery and candidate testing [2]. Slides derived from tissue microarray blocks can be used to study gene amplification and protein overexpression by DNA and RNA *in situ* hybridization or by immunohistochemistry, with tremendous savings on analysis time in addition to labor and reagent costs [3]. The routine use of this technology can accelerate studies seeking the association between molecular changes, clinical endpoints and validation of novel biomarkers [4], as well as in the transition of basic research results to clinical applications [5,6].

However, to address the functional importance of interesting molecules, researchers, before using stored tissues, often turn to cell lines model systems. Although this is a very informative approach, often the selection of the most appropriate cell line(s) is a very difficult choice. Artificial tissue blocks consisting of cell lines (cell microarray, CMA) of different origin, formalin fixed and paraffin embedded, can quickly and comprehensively evaluate the expression of novel proteins or markers and assess their subcellular localization. Cell arrays can become powerful tools to study genomic-scale cell-based gene function analyses [7,8] and are even more powerful with the incorporation of high definition image analysis. 

In stem cell science and particularly for induced pluripotent stem cells, or iPSCs, CMA technology can offer several advantages particularly in screening cell population to search for bonafide clones. iPSs require the introduction of reprogramming factors into mature and fully differentiated cells leading to the drastic epigenetic changes necessary to drive the cells toward an embryonic-like state and re-establish pluripotency. The pluripotent cells can then be differentiated into cells of interest including the three cell lineages required to form the body’s organs, nervous system, skin, muscle and skeleton [9,10,11]. However, each clone (regardless of the number) obtained for each reprogramming event, need to be phenotyped and characterized even if they are partially reprogrammed or aborted. In general, reprogramming is a slow process with poor kinetics, nonetheless the number of clones that generated is usually very high therefore the use of CMA can minimize the time and processing costs associated with the selection and analysis since a whole set of cells can simultaneously be tested for a number of stem biomarkers and molecular parameter associated with iPSCs. 

Here, we describe the application of CMA technology to study iPS clones and to standardize operating procedures in order to make reliable and interpretable cell arrays.

## 2. Experimental Section

### 2.1. Cell Culture for CMA Preparation

The CMA construction requires the initial preparation of cell paraffin blocks [12]. Human AF22 induced pluripotent stem (iPS) cell-derived long term neural stem (lt-NES) cells [13] were cultured according to previously described method [14]. Briefly, cells were maintained in 0.01% Poly-L-ornithine (Sigma, Milan, Italy) and 10 μg/mL laminin (Sigma) coated flask, using DMEM-F/12 medium (Euroclone, Milan, Italy) supplemented with 1% N2 and 0.1% B27 (Life Technologies, Monza, Italy), 10 ng/mL EGF and 10 ng/mL FGF2 (Peprotech EC, London, UK). To induce neuronal differentiation, cells were plated in Neurobasal (Life Technologies) and DMEM-F/12 (Euroclone) 1:1 with 0.5% N2, 1% B27, without EGF and bFGF and cultured for 3 weeks. Cells were passaged every 2 to 3 days using 0.05% trypsin-EDTA solution (Sigma). 

Three testing conditions were used for array construction: (i) cell trypsinization, (ii) mechanical by scraping with a rubber policeman and (iii) growth on coverslips.

(i) *Cell trypsinization*: The cells, 5 × 10^6^, were detached from the plate using trypsin-EDTA (Sigma), washed several times with PBS (Euroclone), and the final cell pellet was fixed in 4% PFA (Electron Microscopy Sciences) at 4 °C for 20 min. The pellet thus formed was resuspended in 300 μL of low melting agarose 3% (Sigma), in 1× PBS at 55 °C and incubated for 30 min on ice. After 24 h incubation in 1% PFA, prior to the paraffin embedding procedure, the agarose pellet was processed in an automatic tissue processor (Fully Enclosed tissue Processor Leica ASP300S, Leica Microsystems, Milan, Italy) for dehydration by incubating the pellet in solutions with increasing alcohol content under vacuum pressure. 

(ii) *Mechanical Scraping*: About 5 × 10^6^ were immediately fixed in 4% paraformaldehyde (PFA) for 20 min, scraped with a rubber policeman and centrifuged 3 min at 300 × g at 4 °C. The fixed cells were gently re-suspended in 300 μL of low melting agarose (3%) in 1× PBS at 55 °C, transferred into a 0.5 mL conical tube and incubated in ice for 30 min. The cell-agarose-pellet was fixed in 1% PFA for 24 h and then embedded in paraffin with an automatic tissue processor. To evaluate an optimal cell concentration and density, 5 μm sections were hematoxylin-eosin stained and were used to select appropriate areas for CMA construction. 

(iii) *Cells grown on coverslip*: AF22-iPS derived cells were grown on small, circular coverslips and placed in wells of a tissue culture plate. After fixation, the cells on coverslips were removed from the wells of the plate for antibody staining [15]. Grown cells were fixed in cold 4% paraformaldehyde for 15 min at room temperature (RT) and washed in PBS. 

### 2.2. CMA Construction

Galileo TMA CK4500 platform (Isenet, Milan, Italy) was used for CMA construction. A hollow needle of 1mm diameter was used to pick cell cores from the donor paraffin block, which were then assembled in the recipient block. Up to 80 consecutive sections of 5 µm thickness can be cut from the CMA block and mounted on microscope slides. The slides were now ready to be assayed with different stemness and differentiation markers. 

### 2.3. Immunofluorescence (IF) Analysis

Cell array slices were deparaffinized and rehydrated by incubating in solutions with decreasing alcohol content. Antigen retrieval was conducted by boiling the sample slides in citrate buffer 0.01 M pH 6. Both coverslip and CMA slices were incubated in 0.1 M glycine for 10 min at RT and then in blocking solution composed by 5% donkey serum, 0.6% Triton in PBS for 30 min at RT. Samples were immunostained at 4 °C overnight in blocking solution with primary antibodies anti-Nestin (1:50; Millipore, Milan, Italy), anti-sox2 (1:300; Millipore), anti-β3-tubulin (1:100; Sigma) and anti-sel1L (5 μg/mL) [16]. For the immunofluorescence characterization, samples were incubated with appropriate secondary antibodies (Rhodamine-Red antimouse IgM and anti-rabbit IgG, Alexa Fluor 488 anti-mouse IgG, (Jackson ImmunoResearch, distributed by Li StarFish, Milan, Italy) and the nuclei were counterstained with Hoechst 33258. The samples were mounted with GelMount aqueous mounting medium (Sigma). The images were acquired using a Leica DMI4000B inverted microscope linked to a DFC360FX or to a DFC280 cameras (Leica Microsystems).

## 3. Results

### 3.1. CMA Technology to Screen for Bonafide Pluripotent Stem Cells and Evaluation of Population Heterogeneity within the Lines

**Figure 1:**
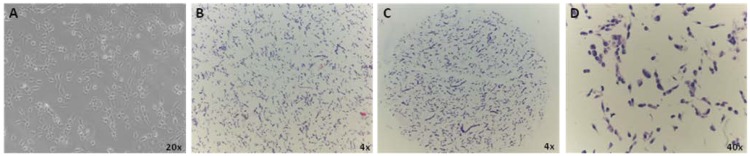
AF22 iPS-derived cells grown on cover slip (**A**); cell density and distribution of paraffin embedded cells in the donor block, using cell scraping and hematoxylin-eosin stain (**B**) and 1mm diameter cell core showing the distribution and architecture of the scraped cells within each core cell microarray (CMA) (**C**) and stained with hematoxylin-eosin (**D**).

CMA is highly informative to immunophenotypically characterize any cell line although it is easier for those cells that grow in suspension since embedding allows the preservation of the 3D structures and the multi-cellular organization. Here, we applied CMA technology to validate the differentiation of the human adherent and neuralized iPS cell model AF22 [13]. We incorporated the AF22 cells into an array to demonstrate the usefulness of this technique and quickly and efficiently assess their differentiation using antigen expression. Harvested cells were prepared and fixed in formalin, suspended in low-melting agarose and embedded in paraffin to produce a cell block. The semi‑automatic tissue microarrayer Galileo CK 4500 was used to remove in triplicate 1 mm-cores from each cell block and to transfer them into a recipient paraffin block at precise coordinates (x; y). The three-dimensional distribution of cells was evaluated by quantifying hematoxylin-stained (HE) 5‑μm sections. Figure 1 shows the distribution AF22 cells grown on coverslip (A), scraping-harvested cells embedded in the donor block (Hematoxylin-eosin, HE, stained, B) and from a selected CMA core slice (C) with subsequent HE staining (D). Image analysis results show that the mean number of cells/mm^2^ in donor block sections (B) is close to those cells cultured on coverslip (A). This is possible since scraping and immediate embedding keeps the cell density intact in addition to good 3D distribution within matrix. Not only the scraping techniques maintains the cells density (A), but also the morphology after CMA-coring (B).

**Figure 2:**
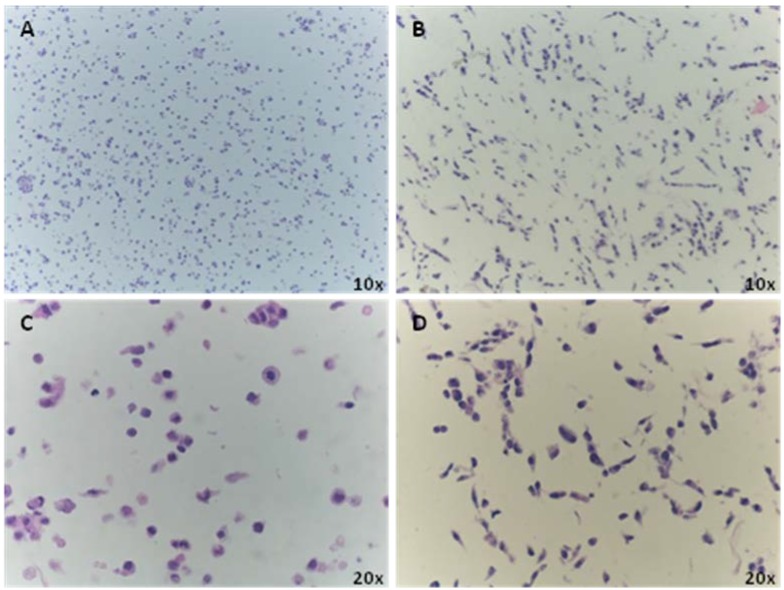
Morphologic differences between trypsinized (**A** and **C**) and scraped cells (**B** and **D**). Images A and C show the cells with a round morphology at different magnifications. The mechanical scraping instead displays a better preservation of cell morphologycompared to those grown on coverslip (Figure 1A), facilitating visualization of the nucleus and cytoplasm staining.

In order to try to preserve cell morphology we assessed two different methodological approaches in cell harvesting: trypsinization and mechanical scraping using a rubber policeman. Figure 2 shows that PFA fixation followed by mechanical scraping preserves both the cell morphology and architecture (B and D); on the other hand trypsin treatment disrupts the elongated cell morphology with consequent decrease in staining visibility (A and C). 

### 3.2. Immunocharacterization of AF22 iPS-Derived Cells by CMA Technology versus Cover Slip

**Figure 3:**
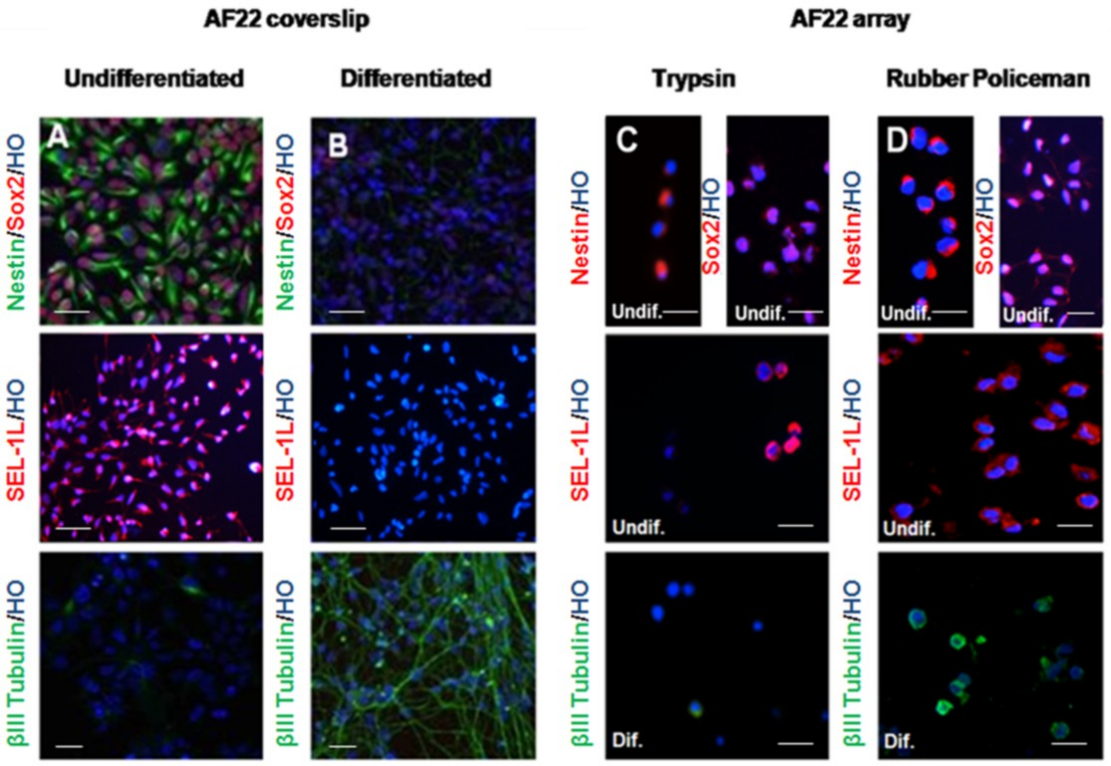
Immunocharacterization of the AF22 iPS-derived cell line by CMA technology *versus* coverslip. Undifferentiated (**A**) and differentiated AF22 cells (**B**) grown on coverslip were analyzed for Nestin, Sox2 and β3-tubulin expression by immunofluorescence. Undifferentiated cells were uniformly immunopositive for the neural precursor cell markers Nestin, Sox2 and SEL1L, a protein involved in neural lineage commitment but negative for the neuronal differentiation markers β3-tubulin. A clear decrease of Nestin, Sox2 and SEL1L is shown in the differentiated (**B**) cells with the concomitant increase of β3-tubulin expression. AF22 cells grown in stemness and differentiated conditions were simultaneously grown in flasks and collected either by trypsinization (**C**) or with a rubber policemen (**D**). Scale bars: 100 µm.

AF22 iPS-derived cell line was induced to differentiate into neuronal lineage for 21 days in three different methodological conditions (coverslip, trypsinization and mechanical scraping as described in the material and method section). Agarose cell pellets were embedded in paraffin and CMA construction was assessed using the Galileo CK4500 platform. IF analysis was performed to investigate the expression and distribution of the stem cell markers Nestin, Sox 2, SEL1L and β3‑tubulin, as previously described (Figure 3) [17]. The mechanical harvesting (3D) of the cells facilitates the immunofluorescence analysis when compared to the enzymatic treatments (3C). Detachment of the cells using a rubber policeman gathers the majority of the cells from the flask maintaining the number of cells and preserving the elongated neuronal morphology. Moreover, this approach allows better marker localization (more similar to coverslip grown cells) if compared to trypsinized cells. These characteristics are very suitable for CMA applications.

## 4. Discussion

In 2005, Ferrer *et al*. and Waterworth *et al.* [7,18] developed and immunophenotyped a panel of paraffin-embedded cell lines using five human adenocarcinoma lines. In 2006, Andersson *et al.* described cell TMAs (cells and tissues) as a tool for antibody-based proteomics using 46 frequently used cell lines in addition to 12 patient cell samples [12]. Therefore, microarrays generated from formalin fixed and paraffin embedded cultured cell lines can serve as a platform for *in vitro* analysis of protein expression profiles. To our knowledge, no “cell microarrays” (CMAs) have yet been reported to analyze antigen expression, by immunohistochemistry, of human pluripotent stem cells to demonstrate the utility of CMAs as a source for the rapid screening of molecules of interest. Yamazoe and Iwata have used CMAs to screen feeder cells in differentiation and candidate cells for specific ES cell differentiation using the PA6-mouse ES cell co-culture system [19]. The rapid and efficient screening of iPS derived cell clones, using CMA technology, is very advantageous considering that each reprogramming event generates a high number of abortive clones [20] all of which need to be molecularly characterized. In this respect, the CMA platform offers several advantages, including minimal consumption of valuable biological samples and reagents, the costs associated with pluripotent stem cells expansion and differentiation are very high. The *in vitro* assembly of cultured iPS cells in a CMA opens up possibilities to analyze not only the expression of stem cell biomarkers but also to analyze the manipulation of the cells prior to fixation, such as check for the presence/absence of the reprogramming vehicle, cell phenotype and heterogeneity, morphological features, shape, nucleus/cytoplasm ratio and number of intracellular components. Fixing harvested cells in agarose molds decreases the number of cells necessary for marker expression analysis, when compared to coverslip growth (Figure 1). This approach is very advantageous considering that the proliferation rate of fully differentiated stem cells is very low [21]. With only one cell pellet a large panel of antigens can be tested since from a single recipient block over a hundred of slices can be sectioned and tested [22]. Moreover on a single recipient block different samples, representing several iPS clones and/or multiple differentiation statues, can be inserted. Thus, TMA technology advantages, such as time-saving in analysis, labor and reagent costs [3], can be effectively translated to cellular samples. Therefore, CMAs can be classified as a highthroughput technique when dealing with iPS derived clones that need to be characterized [20]. 

An important aspect of CMA technology is to preserve cell morphology. Using the AF22 neuralized iPS cell line as a cellular model to validate the method, we investigated two experimental conditions: cell trypsinization and mechanical scraping using a rubber policeman. Here, we show that mechanical scraping is able to maintain cell morphology in a comparable way to those cells grown on coverslip. Indeed trypsin treatment cleaves the binding of anchorage protein to the dish, generating round cells with poor morphology that does not reflect the original one, increasing the possibility to misinterpret immunofluorescence staining (*i.e.*, false negative). Otherwise, mechanical scraping after fixation with PFA enables better preservation of cell morphology and thus nucleus/cytoplasm ratio is maintained, leading to better analysis of the images. The advantages in the application of CMA platform are connected to the precision in the spot definitions and to the traceability of a large number of samples and digital reporting. This automation is highly important when dealing with iPS clones that can be simultaneously analyzed as a whole set of cells, minimizing any possibility of error [23].

Altogether, these results support the use of CMA platform for the screening of large number of cell lines. In particular morphology preservation techniques enhance the power of this technology and introduce CMA as a new tool for stem cell research and specifically iPS clone screening. 
